# Growth and death kinetics of CHO cells cultivated in continuous bioreactor at various agitation rates

**DOI:** 10.1186/1753-6561-5-S8-P101

**Published:** 2011-11-22

**Authors:** Frédérique Balandras, Eric Olmos, Caroline Hecklau, Fabrice Blanchard, Emmanuel Guedon, Annie Marc

**Affiliations:** 1 Laboratoire Réactions et Génie des Procédés, UPR CNRS 3349, Nancy-Université, Vandœuvre-lès-Nancy, France

## Background

Mammalian cells are known to be sensitive to hydrodynamic stresses. Therefore, soft agitation and aeration are generally recommended in culture bioreactor to prevent cell damages. Nevertheless, at the industrial scale, this may induce CO_2_ accumulation, high mixing times, poor air dispersion and concentration gradients. In batch reactor, we have previously found that growth kinetics of CHO cells could be enhanced by increasing stirring frequencies from 80 to 600 rpm. However, these studies were limited in time due to depletion of substrates leading to a cell death which is not associated to hydrodynamic constraints. To overcome the effects on cell death from those nutritional limitations, in this work, longer stationary cultures of CHO cells were carried out in continuous mode in a 2-liter reactor operating with various stirring profiles.

## Materials and methods

CHO 320 cells were grown in a serum-free medium PF-BDM in a sparged and stirred tank reactor (1.4 L working volume; pO_2_: 50%; pH: 7.4; temperature: 37°C). Continuous culture was started in mid-exponential phase (*D*=0.02 h^-1^) and two agitation rate profiles were applied : 300-600-300-600 rpm and 600-300-600-800-900-1000 rpm. Viable and necrotic cell densities were estimated according to the Trypan blue exclusion method. Lysed cells were measured via the LDH release assay, whereas apoptotic cells were analysed by using Guava Easycyte cytometer. Glucose, lactate and glutamine concentrations were assayed with enzymatic commercial kits (Ellitech, Biomerieux, R-Biopharma) and ammonia concentration with a selective probe (Orion). Bioreactor hydrodynamics was numerically simulated by using Computational Fluid Dynamics to calculate the power dissipation rates and the velocity fields at each agitation rate (Fluent 6.3). The experimental validations were performed by Laser Doppler Velocimetry.

## Results

### Continuous culture performed with the first agitation rate profile : 300-600-300-600 rpm

**Figure 1:**
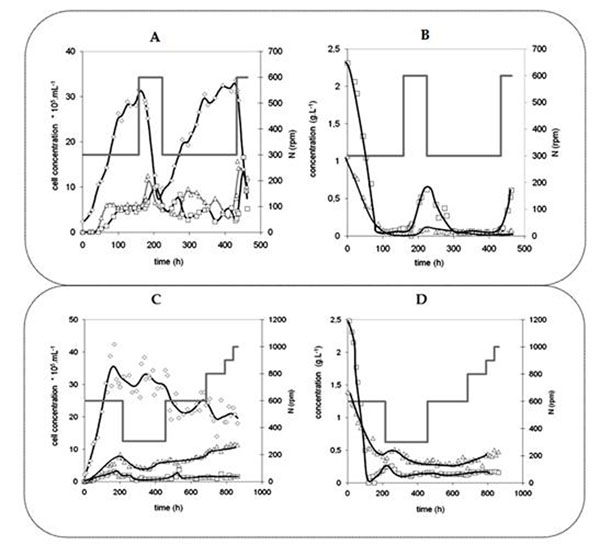
Kinetics of continuous cultures performed with two different profiles of agitation rates (—): (A, C) viable cells (-◇-), dead cells (-□-), lysed cells (-Δ); (B, D) glucose (-□-) and glutamine (-Δ-)

Based on previous batch culture results [1], the batch phase was started at 300 rpm during 48 h in order to obtain the mid-exponential cell growth phase before starting the continuous mode (Figure 1A). Viable cell concentration increased to 30.10^5^ cells.mL^-1^ at the steady state, while dead and lysed cells reached an average level of 5.10^5^ cells.mL^-1^. A step of agitation rate from 300 to 600 rpm induced a 70 % decrease in viable cells and a doubled dead cell concentration. The same variation was obtained for the second step of the experiment (300 rpm to 600 rpm) but with higher dead and lysed cell concentrations of 13.10^5^ cells.mL^-1^. Kinetics of glucose and glutamine consumption were monitored during the culture (Figure 1B). After 100 h of continuous culture, glutamine concentration decreased down to a limiting level around 0.03 g.L^-1^ and remained constant all over the experiment. Following the step from 300 to 600 rpm, glucose concentration increased from 0.1 to 0.6 g.L^-1^ along with the viable cell concentration decrease from 28.10^5^ to 8.10^5^ cells.mL^-^^1^. Consequently, no glucose limitation occurred at 600 rpm. Under hydrodynamic stresses, cell death may occur either by necrosis or apoptosis mechanisms [2,3]. Necrosis is a sudden phenomenon followed by a rapid cell lysis, whereas apoptosis involves enzymatic cascades reactions leading to molecular, enzymatic and structural properties modifications. In our study, analysis of dead cell populations by using flow cytometry revealed that cells mostly died by apoptosis for the first step from 300 to 600 rpm, while necrosis became the predominant death mechanism after the second step (Table 1).

### Continuous culture performed with the second agitation rate profile : 600-300-600-800-900-1000 rpm

For the second run, the batch phase was started at 600 rpm since such an agitation rate was shown not to be deleterious to CHO cells cultivated in batch mode [1]. In this case, the cells reached an average concentration of 30.10^5^ cells mL^-1^ at the steady state of the continuous culture (Figure 1C). However, lysed cells accumulated up to 8.10^5^ cells.mL^-1^ after 200 h of culture. The first change from 600 to 300 rpm did not significantly influence the viable cell density, while a slight decrease of lysed cell concentration was observed. It has to be noted that the increase in the stirring rate from 600 to 1000 rpm did not lead to massive cell death. While the viable cell density decreased and remained at 20.10^5^ cells.mL^-1^, the lysed cell concentration gradually increased to reach 10.10^5^ cells.mL^-1^ and the dead cell density (Trypan blue) remained negligible at 2.10^5^ cells.mL^-1^. In these conditions, no glucose and glutamine limitation was observed (Figure 1D). Dead cell analysis by Guava cytometry indicated a maximal apoptotic cell level around 5 to 10% of total cells throughout the culture (Table 1). Necrotic cells appeared only at 800 rpm and accounted to 40 % of the total cells at 1000 rpm. Then, cell death occurred essentially by lysis and necrosis during this second continuous culture.

**Table 1:** Percentages of apoptotic and necrotic cells measured by using a GUAVA cytometer

Time (h)		RPM		Necrotic cells (%)		Apoptotic cells (%)	
Culture 1	Culture 2	Culture 1	Culture 2	Culture 1	Culture 2	Culture 1	Culture 2

50	50	300	600	7	4	2	6
100	150	300	300	19	2	2	4
150	340	600	300	9	1	15	4
180	480	600	600	2	2	31	7
280	600	300	600	4	2	34	8
320	675	300	800	23	8	8	5
380	745	300	800	21	10	5	8
430	825	600	900	18	30	6	10
450	865	600	1000	49	40	16	13

## Discussion

When CHO cells were cultivated in a continuous mode and subjected to an increase in power dissipation rates from 0.32 (300 rpm) to 2.5 W.kg^-1^ (600 rpm), massive cell necrosis and apoptosis occurred, while this cellular response was not observed in batch mode for the same levels of agitation. Therefore, the CHO cell death could be attributed to the observed glutamine limitation known to induce apoptosis [4]. Furthermore, cells initially cultivated with a higher dissipation rate (600 rpm, 2.5 W.kg^-1^) revealed a better ability to resist to very high dissipation rates (1000 rpm, 11 W.kg^-1^), suggesting cell adaptation or selection. In conclusion, the growth and death mechanisms of CHO cells cultivated at high dissipation rates strongly depend on the sequence of power dissipation levels and on the availability of nutrients. Chemostat could be thus an appropriate system to better understand the complex cellular response to hydrodynamic stresses.
